# 6-(Trifluoro­meth­yl)pyrimidine-2,4(1*H*,3*H*)-dione monohydrate

**DOI:** 10.1107/S1600536810022683

**Published:** 2010-06-18

**Authors:** Gong-Chun Li, Hong-Sheng Wang, Yu-Jiao Niu, Feng-Ling Yang

**Affiliations:** a College of Chemistry and Chemical Engineering, Xuchang University, Xuchang, Henan Province 461000, People’s Republic of China

## Abstract

The title compound, C_5_H_3_F_3_N_2_O_2_·H_2_O, was prepared by the reaction of ethyl 4,4,4-trifluoro-3-oxobutano­ate with urea. In the crystal, the 6-(trifluoro­meth­yl)pyrimidine-2,4(1*H*,3*H*)-dione and water mol­ecules are linked by N—H⋯O and O—H⋯O hydrogen bonds. A ring dimer structure is formed by additional inter­molecular N—H⋯O hydrogen bonds.

## Related literature

For applications of pyrimidine derivatives as pesticides and pharmaceutical agents, see: Condon *et al.* (1993 ▶); as agrochemicals, see: Maeno *et al.* (1990 ▶); as anti­viral agents, see: Gilchrist (1997 ▶); as herbicides, see: Selby *et al.* (2002 ▶).
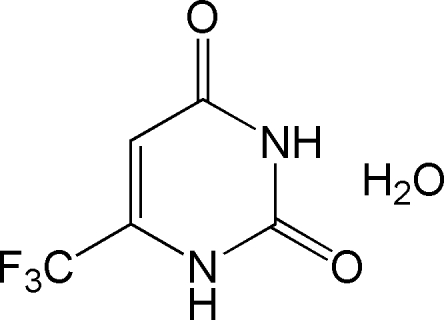


## Experimental

### 

#### Crystal data

C_5_H_3_F_3_N_2_O_2_·H_2_O
*M* *_r_* = 198.11
Monoclinic, 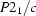

*a* = 5.0250 (8) Å
*b* = 7.046 (1) Å
*c* = 20.769 (2) Å
β = 91.300 (7)°
*V* = 735.16 (17) Å^3^
*Z* = 4
Mo *K*α radiation
μ = 0.19 mm^−1^
*T* = 113 K
0.24 × 0.20 × 0.18 mm

#### Data collection

Rigaku Saturn724 CCD diffractometer
Absorption correction: multi-scan (*CrystalClear-SM Expert*; Rigaku/MSC, 2009 ▶) *T*_min_ = 0.956, *T*_max_ = 0.966
6863 measured reflections
1747 independent reflections
1382 reflections with *I* > 2σ(*I*)
*R*_int_ = 0.029

#### Refinement

*R*[*F* ^2^ > 2σ(*F* ^2^)] = 0.036
*wR*(*F* ^2^) = 0.100
*S* = 1.07
1747 reflections
134 parameters
H atoms treated by a mixture of independent and constrained refinement
Δρ_max_ = 0.33 e Å^−3^
Δρ_min_ = −0.21 e Å^−3^

### 

Data collection: *CrystalClear-SM Expert* (Rigaku/MSC, 2009 ▶); cell refinement: *CrystalClear-SM Expert*; data reduction: *CrystalClear-SM Expert*; program(s) used to solve structure: *SHELXS97* (Sheldrick, 2008 ▶); program(s) used to refine structure: *SHELXL97* (Sheldrick, 2008 ▶); molecular graphics: *CrystalStructure* (Rigaku/MSC, 2009 ▶); software used to prepare material for publication: *CrystalStructure*.

## Supplementary Material

Crystal structure: contains datablocks global, I. DOI: 10.1107/S1600536810022683/fj2309sup1.cif

Structure factors: contains datablocks I. DOI: 10.1107/S1600536810022683/fj2309Isup2.hkl

Additional supplementary materials:  crystallographic information; 3D view; checkCIF report

## Figures and Tables

**Table 1:** Hydrogen-bond geometry (Å, °)

*D*—H⋯*A*	*D*—H	H⋯*A*	*D*⋯*A*	*D*—H⋯*A*
O3—H3*B*⋯O1^i^	0.825 (17)	2.017 (18)	2.7815 (13)	153.9 (17)
O3—H3*A*⋯O2^ii^	0.86 (2)	1.95 (2)	2.8066 (13)	176.0 (17)
N2—H2⋯O3	0.896 (17)	1.824 (17)	2.7191 (14)	177.9 (16)
N1—H1⋯O1^iii^	0.954 (17)	1.896 (18)	2.8490 (14)	176.4 (16)

Symmetry codes: (i) 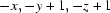; (ii) 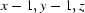; (iii) 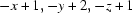.

**Fig. 1.**
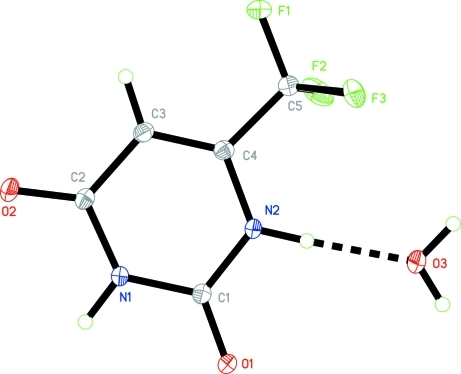
The asymmetric unit of the title compound, (I), with displacement ellipsoids drawn at the 30% probability level.

**Fig. 2.**
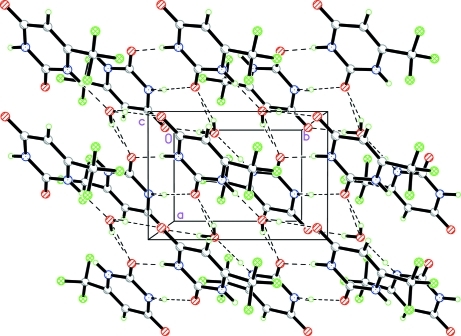
The packing diagram of the title compound. Intermolecular hydrogen bonds are shown as dashed line.

## supplementary crystallographic information

### Comment

Pyrimidine derivatives are very important molecules in biology and have many application in the areas of pesticide and pharmaceutical agents (Condon *et al.*, 1993). For example, imazosulfuron, ethirmol and mepanipyrim have been commercialized as agrochemicals (Maeno *et al.*, 1990). Pyrimidine derivatives have also been developed as antiviral agents, shch as AZT, which is the most widely used anti-AIDS drug (Gilchrist, 1997). Recently, a new series of highly active herbicides of substituted azolylpyrimidines were reported (Selby *et al.*, 2002). In order to discover further biologically active pyrimidine compounds, the title compound, (I), was synthesized and its crystal structure determined (Fig. 1).

In the crystal strucrure, The part of 6-(trifluoromethyl)pyrimidine-2,4(1*H*,3*H*)-dione and water molecule are linked by N—H···O and O—H···O hydrogen bonds. The ring dimer structure is formed by addition intermolecular N—H···O hydrogen bonds.

### Experimental

To 35 ml absolute ethanol sodium (1.38 g, 60 mmol) was added. When sodium was dissppeared, ethyl 4,4,4-trifluoro-3-oxobutanoate(5.50 g, 30 mmol) and urea (1.80 g, 30 mmol) were added to the solution. The mixture was refluxed for 20 hr., The solvent was evaporated *in vacuo* and the residue was washed with water. The title compound was recrystallized from water and single crystals of (I) were obtained by slow evaporation.

### Refinement

All H atoms were placed in calculated positions, with C—H = 0.95 Å, O—H = 0.86 Å or 0.825 Å, and included in the final cycles of refinement using a riding model, with *U*_iso_(H) = 1.2Ueq(C).

### Crystal data

**Table:**

C_5_H_3_F_3_N_2_O_2_·H_2_O	*F*(000) = 400
*M**_r_* = 198.11	*D*_x_ = 1.790 Mg m^−^^3^
Monoclinic, *P*2_1_/*c*	Mo *K*α radiation, λ = 0.71075 Å
*a* = 5.0250 (8) Å	Cell parameters from 2492 reflections
*b* = 7.046 (1) Å	θ = 2.0–27.9°
*c* = 20.769 (2) Å	µ = 0.19 mm^−^^1^
β = 91.300 (7)°	*T* = 113 K
*V* = 735.16 (17) Å^3^	Prism, colorless
*Z* = 4	0.24 × 0.20 × 0.18 mm

### Data collection

**Table:**

Rigaku Saturn724 CCD diffractometer	1747 independent reflections
Radiation source: rotating anode	1382 reflections with *I* > 2σ(*I*)
multilayer	*R*_int_ = 0.029
ω scans	θ_max_ = 27.9°, θ_min_ = 2.0°
Absorption correction: multi-scan (*CrystalClear*-SM Expert; Rigaku/MSC, 2009)	*h* = −6→6
*T*_min_ = 0.956, *T*_max_ = 0.966	*k* = −6→9
6863 measured reflections	*l* = −27→27

### Refinement

**Table:**

Refinement on *F*^2^	Primary atom site location: structure-invariant direct methods
Least-squares matrix: full	Secondary atom site location: difference Fourier map
*R*[*F*^2^ > 2σ(*F*^2^)] = 0.036	Hydrogen site location: inferred from neighbouring sites
*wR*(*F*^2^) = 0.100	H atoms treated by a mixture of independent and constrained refinement
*S* = 1.07	*w* = 1/[σ^2^(*F*_o_^2^) + (0.0589*P*)^2^ + 0.0166*P*] where *P* = (*F*_o_^2^ + 2*F*_c_^2^)/3
1747 reflections	(Δ/σ)_max_ < 0.001
134 parameters	Δρ_max_ = 0.33 e Å^−^^3^
0 restraints	Δρ_min_ = −0.21 e Å^−^^3^

### Special details

**Table:**

Geometry. All e.s.d.'s (except the e.s.d. in the dihedral angle between two l.s. planes) are estimated using the full covariance matrix. The cell e.s.d.'s are taken into account individually in the estimation of e.s.d.'s in distances, angles and torsion angles; correlations between e.s.d.'s in cell parameters are only used when they are defined by crystal symmetry. An approximate (isotropic) treatment of cell e.s.d.'s is used for estimating e.s.d.'s involving l.s. planes.
Refinement. Refinement of *F*^2^ against ALL reflections. The weighted *R*-factor *wR* and goodness of fit *S* are based on *F*^2^, conventional *R*-factors *R* are based on *F*, with *F* set to zero for negative *F*^2^. The threshold expression of *F*^2^ > σ(*F*^2^) is used only for calculating *R*-factors(gt) *etc*. and is not relevant to the choice of reflections for refinement. *R*-factors based on *F*^2^ are statistically about twice as large as those based on *F*, and *R*- factors based on ALL data will be even larger.

### Fractional atomic coordinates and isotropic or equivalent isotropic displacement parameters (Å^2^)

**Table:**

	*x*	*y*	*z*	*U*_iso_*/*U*_eq_	
F1	0.74748 (17)	0.37877 (12)	0.26992 (4)	0.0431 (3)	
F2	0.33139 (17)	0.43344 (12)	0.28202 (4)	0.0433 (3)	
F3	0.54045 (16)	0.25207 (11)	0.34843 (4)	0.0359 (2)	
O1	0.32964 (16)	0.78481 (12)	0.48978 (4)	0.0247 (2)	
O2	1.03039 (17)	0.99065 (12)	0.37275 (4)	0.0261 (2)	
O3	0.09114 (19)	0.33980 (15)	0.43644 (5)	0.0299 (3)	
N1	0.67605 (19)	0.88773 (14)	0.42979 (5)	0.0199 (2)	
N2	0.46193 (19)	0.60395 (14)	0.40583 (5)	0.0187 (2)	
C1	0.4798 (2)	0.76007 (17)	0.44449 (5)	0.0191 (3)	
C2	0.8566 (2)	0.87123 (17)	0.38039 (6)	0.0193 (3)	
C3	0.8191 (2)	0.70430 (17)	0.34028 (6)	0.0196 (3)	
H3	0.9296	0.6828	0.3045	0.023*	
C4	0.6262 (2)	0.58089 (16)	0.35448 (5)	0.0183 (3)	
C5	0.5641 (2)	0.40964 (18)	0.31352 (6)	0.0237 (3)	
H1	0.681 (3)	0.999 (2)	0.4561 (9)	0.048 (5)*	
H2	0.340 (3)	0.518 (2)	0.4169 (8)	0.041 (5)*	
H3A	0.081 (3)	0.234 (3)	0.4166 (10)	0.049 (5)*	
H3B	−0.024 (3)	0.336 (3)	0.4642 (8)	0.043 (5)*	

### Atomic displacement parameters (Å^2^)

**Table:**

	*U*^11^	*U*^22^	*U*^33^	*U*^12^	*U*^13^	*U*^23^
F1	0.0459 (5)	0.0380 (5)	0.0467 (5)	−0.0137 (4)	0.0277 (4)	−0.0219 (4)
F2	0.0400 (5)	0.0422 (6)	0.0467 (5)	0.0051 (4)	−0.0173 (4)	−0.0201 (4)
F3	0.0481 (5)	0.0177 (4)	0.0423 (5)	−0.0059 (4)	0.0095 (4)	−0.0025 (3)
O1	0.0267 (5)	0.0250 (5)	0.0229 (4)	−0.0091 (4)	0.0097 (4)	−0.0050 (3)
O2	0.0237 (5)	0.0225 (5)	0.0325 (5)	−0.0076 (4)	0.0092 (4)	−0.0012 (4)
O3	0.0307 (5)	0.0248 (6)	0.0347 (6)	−0.0110 (4)	0.0140 (4)	−0.0049 (4)
N1	0.0204 (5)	0.0192 (6)	0.0204 (5)	−0.0058 (4)	0.0042 (4)	−0.0025 (4)
N2	0.0191 (5)	0.0165 (5)	0.0208 (5)	−0.0046 (4)	0.0043 (4)	−0.0009 (4)
C1	0.0190 (6)	0.0193 (6)	0.0190 (5)	−0.0028 (4)	0.0014 (4)	0.0000 (5)
C2	0.0176 (5)	0.0187 (6)	0.0215 (6)	−0.0006 (5)	0.0023 (4)	0.0028 (4)
C3	0.0192 (6)	0.0195 (7)	0.0201 (6)	0.0010 (5)	0.0039 (4)	0.0010 (4)
C4	0.0185 (5)	0.0174 (6)	0.0191 (6)	0.0019 (4)	0.0013 (4)	0.0007 (5)
C5	0.0227 (6)	0.0216 (7)	0.0270 (6)	−0.0019 (5)	0.0064 (5)	−0.0038 (5)

### Geometric parameters (Å, °)

**Table:**

F1—C5	1.3245 (14)	N1—H1	0.954 (17)
F2—C5	1.3374 (15)	N2—C1	1.3636 (15)
F3—C5	1.3328 (15)	N2—C4	1.3729 (14)
O1—C1	1.2317 (14)	N2—H2	0.896 (17)
O2—C2	1.2255 (14)	C2—C3	1.4512 (17)
O3—H3A	0.86 (2)	C3—C4	1.3400 (17)
O3—H3B	0.825 (17)	C3—H3	0.9500
N1—C1	1.3742 (15)	C4—C5	1.5050 (17)
N1—C2	1.3898 (15)		
			
H3A—O3—H3B	105.8 (17)	C4—C3—C2	119.01 (11)
C1—N1—C2	126.37 (10)	C4—C3—H3	120.5
C1—N1—H1	114.8 (11)	C2—C3—H3	120.5
C2—N1—H1	118.8 (11)	C3—C4—N2	123.00 (11)
C1—N2—C4	121.34 (10)	C3—C4—C5	122.52 (11)
C1—N2—H2	115.5 (11)	N2—C4—C5	114.42 (10)
C4—N2—H2	123.2 (11)	F1—C5—F3	107.89 (10)
O1—C1—N2	122.04 (10)	F1—C5—F2	107.48 (10)
O1—C1—N1	122.15 (11)	F3—C5—F2	106.44 (10)
N2—C1—N1	115.80 (10)	F1—C5—C4	112.30 (10)
O2—C2—N1	121.15 (11)	F3—C5—C4	112.34 (10)
O2—C2—C3	124.46 (11)	F2—C5—C4	110.10 (10)
N1—C2—C3	114.39 (10)		
			
C4—N2—C1—O1	178.24 (10)	C2—C3—C4—C5	176.73 (10)
C4—N2—C1—N1	−1.87 (16)	C1—N2—C4—C3	2.53 (18)
C2—N1—C1—O1	179.13 (11)	C1—N2—C4—C5	−174.89 (10)
C2—N1—C1—N2	−0.76 (17)	C3—C4—C5—F1	10.94 (17)
C1—N1—C2—O2	−177.49 (11)	N2—C4—C5—F1	−171.62 (10)
C1—N1—C2—C3	2.59 (16)	C3—C4—C5—F3	132.78 (12)
O2—C2—C3—C4	178.20 (11)	N2—C4—C5—F3	−49.78 (14)
N1—C2—C3—C4	−1.88 (16)	C3—C4—C5—F2	−108.79 (13)
C2—C3—C4—N2	−0.49 (18)	N2—C4—C5—F2	68.65 (13)

### Hydrogen-bond geometry (Å, °)

**Table:**

*D*—H···*A*	*D*—H	H···*A*	*D*···*A*	*D*—H···*A*
O3—H3B···O1^i^	0.825 (17)	2.017 (18)	2.7815 (13)	153.9 (17)
O3—H3A···O2^ii^	0.86 (2)	1.95 (2)	2.8066 (13)	176.0 (17)
N2—H2···O3	0.896 (17)	1.824 (17)	2.7191 (14)	177.9 (16)
N1—H1···O1^iii^	0.954 (17)	1.896 (18)	2.8490 (14)	176.4 (16)

Symmetry codes: (i) −*x*, −*y*+1, −*z*+1; (ii) *x*−1, *y*−1, *z*; (iii) −*x*+1, −*y*+2, −*z*+1.
